# Why the worsening at rest and worsening at night criteria for Restless Legs Syndrome are listed separately: review of the circadian literature on RLS and suggestions for future directions

**DOI:** 10.3389/fneur.2023.1153273

**Published:** 2023-04-27

**Authors:** Arthur S. Walters, Phyllis C. Zee

**Affiliations:** ^1^Department of Neurology, Division of Sleep Medicine, Vanderbilt University Medical Center, Nashville, TN, United States; ^2^Division of Sleep Medicine, Department of Neurology and Center for Circadian and Sleep Medicine, Northwestern University Feinberg School of Medicine, Chicago, IL, United States

**Keywords:** circadian, Restless Legs Syndrome (RLS), periodic limb movements in sleep (PLMS), periodic limb movements while awake (PLMW), polysomnography (PSG), suggested immobilization test (SIT), actigraphy, CLOCK genes

## Abstract

The field of circadian research on Restless Legs Syndrome (RLS) and periodic limb movements (PLMs) is reviewed in general. RLS has five obligatory criteria for diagnosis: (1) an urge to move the legs often accompanied by uncomfortable leg sensations; (2) symptoms are worse at rest, i.e., lying or sitting; (3) there is a least partial and temporary relief of symptoms by activity, e.g., walking or stretching or bending the legs; (4) symptoms are worse later in the day or at night; and (5) mimics of RLS such as leg cramps and positional discomfort should be excluded by history and physical. In addition, RLS is frequently accompanied by PLMs, either periodic limb movements of sleep (PLMS) as determined by polysomnography or periodic limb movements while awake (PLMW) as determined by the suggested immobilization test (SIT). Since the criteria for RLS were based upon clinical experience only, an early question after the development of the criteria was whether criteria 2 and 4 were the same or different phenomena. In other words, were RLS patients worse at night only because they were lying down, and were RLS patients worse lying down only because it was night? Early circadian studies performed during recumbency at different times of the day suggest that the uncomfortable sensations, PLMS, and PLMW as well as voluntary movement in response to leg discomfort follow a similar circadian pattern with worsening at night independent of body position and independent of sleep timing or duration. Other studies demonstrated that RLS patients get worse when sitting or lying down independent of the time of day. These studies as a whole suggest that the worsening at rest and the worsening at night criteria for RLS are related but separate phenomena and that criteria 2 and 4 for RLS should be kept separate based upon the circadian studies, as had been the case previously based upon clinical grounds alone. To more fully prove the circadian rhythmicity of RLS, studies should be conducted to see if bright light shifts the signs and symptoms of RLS to a different circadian time in concert with circadian markers.

## Introduction

This is a narrative review of the circadian rhythmicity of restless legs syndrome (RLS) and periodic limb movements in sleep (PLMS).

Circadian refers to an endogenous and self-sustaining biological rhythm with a period of ~24 h that can be synchronized to an environmental cycle. A diurnal rhythm is a rhythmic physiological process or behavior that is more likely to peak or occur during the daytime. Although some of the studies in this review labeled as “circadian” are more truly “diurnal” in design, we have maintained the original terminology of the authors in all cases. In other words, when authors describe their studies as “circadian” we have used that term and when authors describe their studies as “diurnal” we have used that term.

RLS has five obligatory criteria for diagnosis: (a) an urge to move the legs often accompanied by uncomfortable leg sensations; (b) symptoms are worse at rest, i.e., lying or sitting; (c) there is a least partial and temporary relief of symptoms by activity, e.g., walking or stretching or bending the legs; (d) symptoms are worse later in the day or at night; and (e) mimics of RLS such as leg cramps and positional discomfort should be excluded by history and physical (1). In addition, RLS is frequently accompanied by involuntary movements such as periodic limb movements of sleep (PLMS) as determined by polysomnography or periodic limb movements while awake (PLMW) as determined by the suggested immobilization test (SIT) (1–4). Since the criteria for RLS were developed empirically based on clinical experience alone, an early question was whether criteria (b) and (d) were the same or different phenomena. This was addressed by early circadian studies. A summary of these and other studies follows.

## Circadian studies of the primary sensory and motor features of Restless Legs Syndrome and of periodic limb movements of sleep

In the SIT test, subjects are asked to lie down while awake and are kept at 45 degrees for between 30 and 90 min (1–4). In the traditional SIT, patients are asked to lie perfectly still. The severity of leg sensations can be documented on a visual analog scale filled out by the subjects every 5–20 min during the 30–90 min SIT, and PLMW can be measured continuously during the SIT by actigraphy, EMG, or both (1–7). The 30–90 min SIT tests can be repeated periodically throughout the circadian day during wakefulness.

Our initial two circadian studies were performed in order to assess the contribution that body position, time of day, sleep, wake, and sleep deprivation had on the symptoms of RLS. In our first circadian study, eight patients with idiopathic RLS (mean age 60.4 years, 5 F, 3 M) were entered into the study. We measured the severity of leg sensations and PLMW by SIT tests performed periodically during wakefulness and PLMS was monitored continuously in sleep by polysomnography (5). Data were collected during wakefulness, during sleep, during a night of sleep deprivation, and during the day following the night of sleep deprivation. Core body temperature as a marker of the circadian phase was monitored continuously throughout the entire study.

In our second circadian study, nine subjects with idiopathic RLS (mean age 59.8 years, range 33–72, years, 5F, 4 M, mean duration of RLS symptoms 14.9 years) were entered into the study. For this study, we developed a modified SIT test in which patients could move during the SIT but only in response to leg discomfort. The experimental design was otherwise similar with leg discomfort measured on a visual analog scale during the SIT and PLMS measured continuously during sleep by polysomnography. Similar to the first study, data were collected during wakefulness, during sleep, during a night of sleep deprivation, and during the day following the night of sleep deprivation; core body temperature as a marker of the circadian phase was monitored continuously throughout the entire protocol (6). Thus, in the first circadian study, leg movement recording was used during the SIT to measure involuntary movements (PLMW), whereas in the second circadian study, leg movement recording was used during the SIT to measure voluntary activity in response to the need to move to eliminate leg discomfort (5, 6). In both studies, the results were similar. Sleep, drowsiness, and sleep deprivation had some impact on sensory symptoms and periodic movements. However, uncomfortable sensations, PLMS, and PLMW as well as voluntary movement in response to leg discomfort all followed a clear underlying circadian pattern with worsening at night independent of sleep or sleep deprivation (5, 6). The response was also independent of rest, i.e., lying or sitting, as all testing at different times of day was done in the recumbent position. Symptoms and signs occurred in concert with the declining portion of the circadian core temperature curve. Together, these results indicate that the worsening at rest and the worsening at night criteria for RLS are likely differentially regulated and that the original clinical decision to list criteria (b) and (d) as separate for RLS is correct.

Michaud et al. validated our results (7). Seven patients with idiopathic RLS (mean age 43.9 years, range 25–63 years, 4 F, 3 M) and seven age and sex-matched controls were entered into the study. The authors employed a 28-h modified constant routine. Similar to our studies, leg discomfort was measured every 5 min on a visual analog scale and PLMW was measured continuously during 40-min SIT tests repeated every 2 h in wakefulness. The circadian phase was determined by salivary melatonin and continuous core body temperature. Although leg discomfort was worse in RLS patients than controls, the leg discomfort and PLMW for both groups followed a significant circadian pattern with worsening at night (*P* < 0.01). Unlike our studies, Michaud et al.'s study did not include a night of sleep and therefore did not record PLMS (5–7). However, Michaud et al.'s study had the advantage of employing a control group which our studies did not include (5–7). The results of the Michaud et al.'s study (7) were also slightly different from those of our studies (5, 6) in that the peak of RLS symptoms occurred at the nadir of the core body temperature curve in the Michaud et al. study (7) as opposed to the falling limb of the core body temperature curve in our studies (5, 6). Michaud et al. state that “This small discrepancy between studies is most likely attributable to the difference in the temporal resolution of symptoms assessment (2 h in this study compared with 3 or 4 h in the two aforementioned studies)” (7).

In a second study, Michaud et al. further analyzed the data from their first study (8). They analyzed the severity of leg discomfort at 10, 20, 30, and 40 min within each of the fourteen 40 min SITs administered every 2 h during the 28-h constant routine. They showed that the severity of RLS leg discomfort increased from 10–20 to 30–40 min but only during SITs 7, 8, 9, 10, and 12. This corresponds to the time between 9:20 p.m. and 8:00 a.m., which is the time of night of maximal RLS severity as determined by the previous circadian rhythm studies of RLS (5–7). These results cannot be explained by only a circadian night-time effect or only a worsening at rest effect. If this were strictly a circadian effect, we would not expect to find that the RLS symptoms would progress over the small amount of circadian time encompassed in a short 40-min SIT. On the other hand, if this were strictly a worsening at rest effect, we would not expect that the symptoms would progress during the night time SITs and not the daytime SITs. Thus rest, i.e., lying or sitting, makes RLS worse; however, the worsening at rest is also under the circadian influence.

PLMS are a significant marker of RLS, and it occurs in 80% of patients with RLS. PLMS have been previously noted to mostly occur in stages 1 and 2 of N-REM sleep. The circadian rhythmicity of PLMS was further documented in a study by Duffy et al. (9). The authors studied four adults without RLS (mean age 63.3 years, range 56–78 years, 1 F, 3 M) and with a PLMS index > 20 in an in-patient forced desynchrony protocol with an imposed sleep–wake cycle of 20 h for 12 nights to allow for a separation of circadian and sleep homeostatic influences on leg movements. Time in bed was 6.67 h and bedtimes were progressively advanced in relation to a normal 24-h day. PLMS were detected by polysomnography. Core body temperature was recorded continuously. Plasma melatonin was also measured every 60 min. PLMS in stage 2 sleep were then analyzed. PLMS in stage 2 showed a significant time-within-sleep pattern with the majority of PLMS occurring at the beginning of scheduled sleep episodes (*p* < 0.0001). PLMS also demonstrated a significant circadian rhythm (*p* < 0.0001). PLMS peaked at the circadian phase just before the peak in endogenous melatonin. This peak in PLMS also corresponded to the circadian phase corresponding to just before what would be the habitual bedtime under normal entrained conditions (9).

## Circadian genes and Restless Legs Syndrome/periodic limb movements in sleep

Two studies have shown that RLS is associated with the CLOCK gene which plays a primary role in the generation of circadian rhythms of all types (10, 11). One additional study has shown that PLMS are also associated with CLOCK (12).

In the first study, RLS patients with schizophrenia were found to have this association. The authors studied 190 patients with schizophrenia on antipsychotics (mean age 39.6 years, range 22–66 years) and divided them into 44 subjects with RLS and 146 subjects without RLS. The authors found a significant difference in genotype (*P* = 0.031) and allele carrier frequencies (*p* = 0.010) between the two groups for the CLOCK gene (rs2412646). The distribution of CLOCK haplotypes (rs2412646-rs1801260) was also significantly different between the two groups (*p* = 0.021). The authors suggest that CLOCK gene polymorphisms may predispose subjects with schizophrenia to RLS (10).

In the second study, RLS in non-schizophrenic patients was also found to be associated with CLOCK gene variants. In total, 227 patients with idiopathic RLS (mean age 49.45 years, 153 F, 74 M) and 229 age- and sex-matched controls were studied. Because the allelic frequencies of CLOCK rs1801260 showed marginally significant differences between the two groups, the authors proceeded with a haplotype analysis of rs1801260-rs2412646, which showed an overall significant difference of *p* = 0.013. The non-RLS controls had significantly higher frequencies of the G allele of CLOCK rs1801260 and the G-T haplotype of CLOCK (rs1801260-rs2412646) than in RLS. The authors suggest that these variations may have a protective effect against the development of RLS symptoms (11).

In a third study, the PLMS index (PLMI) was found to be associated with the CLOCK gene. In total, 360 subjects (mean age 57.4 years, range 21–92 years, 119 F, 241 M) were recruited who had undergone overnight polysomnography for clinical reasons and thus served as a convenience sample for the study. When subjects with an apnea–hypopnea index (AHI) > 15 were eliminated, the PLMI was greater among those with the C allele of CLOCK rs1801260 (*p* < 0.05). No such associations were found for RLS in this particular study when all four obligatory criteria for RLS were strictly applied (12).

## Circadian rhythm of central nervous system hyperexcitability in the Restless Legs Syndrome

Numerous studies have documented central nervous system hyperexcitability in RLS (13). However, most of these studies have been performed at a single time point in the day. These observations have been strengthened by the observation that PLMS persist in the presence of total spinal cord transection (13). Thus, it is hypothesized that PLMS are promulgated by a lower spinal cord generator that is normally inhibited by descending spinal cord tracts originating from the cortex or brain stem. The absence of influences from these tracts results in a supraspinal disinhibition. However, unlike the situation in spinal cord transection, RLS patients do not exhibit muscle weakness nor do they have increased muscle tone, both prominent features of spinal cord transection, when the corticospinal tracts are involved. Therefore, aberrations in descending pathways other than the corticospinal tracts must be responsible for supraspinal disinhibition (13).

There have been a few studies, however, that took the time of day into consideration in their assessments. In a study of 11 patients with idiopathic RLS (mean age 50.2 years, 5 F, 6 M) and eight age- and sex-matched controls, transcranial magnetic stimulation (TMS) was given to the area of the motor cortex corresponding to the hand and recording was done from the contralateral first dorsal interosseous muscle (14). The stimulation was done both during the day when there were no RLS symptoms and late at night when RLS symptoms were at their maximum. At night, cortical silent periods tended to shorten and motor thresholds tended to decrease in the RLS group when the night was compared to the day within the RLS group, whereas in the controls they tended to increase at night when the night was compared to day within the control group. Measured parameters were similar between the two groups during the day. However, at night, active motor thresholds were statistically significantly lower in the RLS group compared to controls (*p* = 0.006) (14). These results suggest that the hyperexcitability of the central nervous system in RLS is under the circadian influence.

The blink reflex was also examined by the same group of investigators in the same patient group under the same conditions (15). The R2 recovery was highest during the evening compared to the day in the RLS group, whereas the R2 recovery decreased during the night compared to the day in controls. There was a tendency for R2 recoveries with interstimulus intervals of 300 and 500 ms to be higher at night-time in RLS patients than in the controls, whereas the two groups showed similar results in the daytime. The results are again compatible with the circadian modulation of central nervous system hyperexcitability in RLS (15).

A separate group looked at the circadian variability of spinal cord reflexes in RLS patients vs. controls in three separate studies (16–18). In the first study standard, electromyographic (EMG) techniques were used to quantify patellar reflexes in 11 patients with idiopathic RLS (mean age 48.1 years, 7 F, 4 M, mean duration of RLS symptoms 18.5 years) and nine age- and sex-matched controls during both the night and the following day (16). An analysis of digital camera footage was used to measure knee angular velocity and displacement for the patellar reflex. RLS patients had a statistically significantly attenuated quadriceps EMG amplitude during patellar reflex testing in the evening compared to the morning (*p* = 0.0078). RLS patients also had a statistically significantly attenuated quadriceps EMG amplitude during patellar reflex testing in the evening compared to evening measurements in the control group (*p* = 0.040). In addition, when the digital camera footage was reviewed, the RLS patients also had statistically significantly less knee angular displacement in the evening compared to the control group (*p* = 0.018). Thus, circadian rhythmicity of the patellar reflex was demonstrated in RLS patients but the results went in the opposite direction from that which was expected and the results were, therefore, not in support of the central nervous system hyperexcitability hypothesis for RLS (16).

A second study by the same group examined the plantar reflex in 13 patients with idiopathic RLS (mean age 45.5 years, 9F, 4M, mean duration of RLS symptoms 16.6 years) and 13 age- and sex-matched controls (17). As in the previous study, the reflex response was assessed electromyographically and by a review of camera footage, and, as previously, the protocol was performed both in the evening and the morning. Within the RLS group, evening ankle changes were statistically significantly larger and faster in the evening compared to the morning. No such variation was noted in the controls. However, the RLS patients had a significantly smaller change in ankle angle and significantly slower ankle movements in both the evening and the morning compared to controls (*p*-values ranged from 0.01 to 0.04). In addition, the maximum amplitude of the lateral gastrocnemius muscle was lower in the RLS patients than the controls in the morning (*p* = 0.04). Thus, the results of the second study are in support of circadian modulation of the plantar reflex in RLS but only partially in support of the central nervous system hyperexcitability hypothesis for RLS (17).

A third study by the same group examined the flexor withdrawal and crossed extensor reflexes in 12 patients with idiopathic RLS (mean age 45.8 years, 8 F, 4 M, mean duration of RLS symptoms of 16.7 years) and 12 age and sex matched controls with a similar protocol to the first two studies (18). For ankle angle and velocity, both of the reflexes showed a circadian rhythm in RLS patients but not in controls, and, within the RLS group, the reflex changes were significantly larger and faster in the evening than in the morning. However, except for the additional finding that the hallux angle was greater in RLS patients in the evening than in the morning during the flexor withdrawal reflex, no other significant changes were found among the many variables measured (18). Overall, however, a circadian rhythmicity of the flexor withdrawal and crossed extensor reflexes is suggested within the RLS group and the results are, in general, supportive of the central nervous system hyperexcitability hypothesis of RLS. Within the RLS group, the hyperexcitability was demonstrated in the evening when RLS symptoms are expected to be maximum (18).

## Circadian rhythm of peripheral nervous system hyperexcitability in the Restless Legs Syndrome

Studies in RLS performed at a single point in time are also supportive of peripheral nerve hyperexcitability in RLS (13). Two studies have examined the circadian variability of peripheral nerve hyperexcitability in RLS (19, 20). In these studies, the authors employed the current perception threshold test (CPT) where the toes were stimulated by a constant alternating current at three different frequencies-−5, 250, and 2,000 Hz. At each of these frequencies, the intensity of the stimulus was varied. The intensity for each frequency was increased until the subject could feel a stimulus. The procedure was then repeated at progressively lower intensities until the subject could no longer feel the stimulus. This then was defined as the lowest detection threshold for that particular frequency (19). In total, 13 patients with idiopathic RLS (mean age 50.5 years, 22 F, 8 M, mean duration of RLS symptoms 10.1 years) and 30 age and sex matched controls were entered into the study and the procedure was administered in the daytime when patients were asymptomatic from RLS symptoms and in the evening when patients were symptomatic from RLS. The RLS patients showed a circadian variability in their response to the stimuli, and in all three frequencies, the patients showed significantly lower mean CPT values at 5, 250, and 2,000 Hz in the evening compared to the morning within the RLS group. When RLS patients were compared to controls, the RLS patients had significantly lower CPT thresholds in the evening compared to controls in the evening at 5 Hz (*p* = 0.02). There were no differences in CPT thresholds between RLS and controls during the day. The 5 Hz frequency is selective for slow pain-related C fibers. These results are reflective of a hyperexcitable peripheral nervous system in RLS and a hyperesthetic state in RLS that is relatively selective for C fibers. This hyperesthesia is worse at night when RLS symptoms are at their maximum. However, since there was a difference between the evening and the morning results within the RLS group for 250 Hz frequency and the 2,000 frequency as well as the 5 Hz frequency, there is also a suggestion that A-delta fibers (250 Hz) and A-Beta fibers (2,000 Hz) may be involved as well. Since the patients had nerve conductions studies to rule out peripheral neuropathy, the authors suggest that the hyperesthesia is centrally mediated in RLS. Furthermore, this hyperesthesia in RLS appears to be under circadian control.

The second study employed a protocol specifically to probe the circadian rhythmicity of the A-delta fibers in 11 patients with idiopathic RLS (mean age 53.2 years, range 22–77 years, 4 F, 7 M) and 11 age and sex matched controls (20). CO_2_ laser pulses were delivered to the hands and feet both during the day and at night. Subjects rated pain in response to the stimulus on a visual analog scale and the sensory threshold intensity was determined as in the aforementioned study (19). The sensory threshold intensity was defined as the lowest stimulus intensity eliciting a distinct pinprick sensation. The results of the visual analog data were analyzed. Cortical laser-evoked potentials (LEPS) produced by the painful stimulus were recorded by scalp EEG, and the N1 potentials and the N2–P2 complex potentials were analyzed. The N1 response is thought to be linked to the sensory-discriminative component of pain and the P2 component of the N2–P2 response is thought to be linked to the attention–affective–emotional aspect of the pain experience. In the RLS group, compared to controls, there was a significantly greater visual analog pain scale rating after foot as well as hand stimulation during the night (*p* = 0.009 and *p* = 0.005, respectively). The night/day ratio of the N1 amplitude and the night/day ratio of the N2–P2 amplitude after foot stimulation were increased in patients compared to controls (*p* = 0.016 and *p* = 0.003, respectively). These results indicate hyperexcitability of the afferent A-delta peripheral nerve fibers in RLS. In addition, a circadian rhythm of the response of A-delta peripheral nerve fibers is demonstrated in RLS with greater sensitivity to painful sensations in the evening when RLS leg discomfort usually peaks (20).

It is not surprising that there is peripheral nervous system hyperexcitability in idiopathic RLS and even more so in the evening. RLS symptoms generated by peripheral neuropathy are identical to those in the idiopathic form and RLS associated with peripheral neuropathy shows an identical circadian variation to that of the idiopathic form with worsening of RLS symptoms in the evening (19, 20). How the hyperexcitability of the central nervous system coordinates with the hyperexcitability of the peripheral nervous system to produce the symptoms of RLS is not completely understood and is the subject of an ongoing investigation, the scope of which is beyond the purview of this review.

## Circadian rhythm of cognition in the Restless Legs Syndrome

Cognition has been studied in RLS. Results have been contradictory and, again, studies have not looked at variability over the circadian day. Two studies have attempted to address this gap. In a study of 33 patients with idiopathic RLS (mean age 65.21 years, 25F, 8M) and 33 age- and sex-matched controls, cognition was tested during both the day and the night with a flanker interference test and with simultaneous EEG, which recorded event-related potentials (ERPs) during the flanker interference test. As a third measure, sLORETA (standardized low-resolution brain electromagnetic tomography) was also employed during the flanker interference test (21). In the flanker interference test, a picture of three arrows arranged on top of each other is shown to the subject. The top and bottom arrows point in the same direction and they can either both point to the right or to the left. The subject is asked to determine the direction of the middle arrow which can point in the same direction (compatible condition) or the opposite direction (incompatible condition) as the top and bottom arrows. The picture is shown for only 300 ms. The subjects indicate their response by pressing a button on their left or right. Multiple pictures are presented sequentially and then the number of correct and incorrect responses is calculated. RLS patients made more errors on the flanker interference test in the evening compared to the morning but this was not true of controls. For the within the same group comparisons of the electrophysiologic data, the event-related N1 potential was greater in the evening than the morning for controls but not for RLS patients. When the two groups were directly compared, N1 was larger in the evening in the controls in the incompatible condition compared to RLS patients. In addition to its relation to the sensory-discriminative aspect of pain, the N1 potential is thought to be related to attentional selection processes such as focusing on task-relevant stimuli (20, 21). When the brain electromagnetic tomography was analyzed, it was shown that this difference between the control group and the RLS patients in the incompatible condition in the evening was found to be rooted in the extra-striate visual cortex. The extra-striate visual cortex plays a role in selecting important information and filtering out irrelevant information. Thus, there is a circadian variability in attentional selection processes in RLS with a worse performance at night. From the experimental protocol employed, this effect seems to be independent of RLS severity, fatigue, measured sleep quality, or medication use.

Instead of focusing on the attentional aspects of cognition, a second study attempted to address the diurnal aspects of the non-attentional matrix of cognition in RLS patients and controls by examining the Default Mode Network (DMN) (22). The DMN consists of connections between discrete areas of the medial and lateral parietal, medial prefrontal, and medial and lateral temporal cortices (23). A major feature of the network is that it is active when the individual is not focused on the outside world. Its major activity occurs in support of emotional processing, self-referential mental activity, and recollection of prior experiences (23). Activities such as daydreaming or reverie or mind wandering occur with activation of the default mode network. It is also active when the individual is thinking about others, thinking about themselves, remembering the past, and planning for the future. In a study of 15 drug-naïve patients with idiopathic RLS (mean age 57.40 years, 9F,6M, mean duration of RLS symptoms 10.33 years) and 15 age- and sex-matched controls fMRI scans were done in the morning and evening. The DMN connectivity patterns were then compared. For each anatomical region of interest, the degree of connection to the other regions of interest was analyzed. For RLS patients, compared to controls, bilateral thalami in RLS patients showed increased connectivity in the morning and decreased connectivity in the evening. For RLS patients, compared to controls, the reverse was true of other regions as the right medial frontal gyrus, left posterior cingulate, and left precuneous since RLS patients showed decreased connectivity in the morning and increased connectivity in the evening. The severity of RLS, as measured by the total IRLS score, the symptom severity subscale, and the quality-of-life subscale of the IRLS was negatively correlated with the connectivity strength of the thalamus in the morning in the RLS group. Thus, there is a diurnal modulation of the DMN in RLS patients. Although the DMN is a cognitive network, the authors propose an interaction between the timing of these cognitive factors and the time of appearance of RLS symptoms. The thalamus, in particular, showed strong connectivity in the RLS patients in the morning and the thalamus is the major way that the abnormal sensations in RLS are relayed to the cortex for conscious appreciation of leg discomfort. Furthermore, this strong connectivity in the thalamus in the morning corresponds to the period of decreased RLS symptomatology and negatively correlates with RLS severity as a whole.

## Dopamine and circadian variation in RLS

Dopaminergic agents are one of the principal therapies for RLS. Three studies looked at circadian variation in the dopaminergic system in regard to RLS (24–26). In dopamine metabolism, L-Tyrosine is converted to L-Dopa by tyrosine hydroxylase with Tetrahydrobiopterin (BH4) as an enzymatic co-factor. L-DOPA is mostly converted to dopamine but a minority is converted to 3-ortho- methyl DOPA (3-OMD). Dopamine is metabolized to dihdroxyphenylacetic Acid (DOPAC) and homovanillic acid (HVA). In a parallel monamineric pathway, serotonin is metabolized to 5-hyroxyindoleacetic acid (5-HIAA). In one of the studies, the circadian variation in dopamine metabolism was examined in the cerebrospinal fluid in 30 medication-free patients with idiopathic RLS (mean age 62.9 years) and 22 age- and sex-matched controls (24). Samples were collected at 10 p.m. and 10 a.m. There was a non-significant variation in dopamine metabolism in controls when the night was compared to day within the control group, but there was a significant variation in dopamine metabolism in RLS subjects when the night was compared to day within the RLS group with larger values in the morning compared to the night for BH4, the HVA/5-HIAA ratio, and 3-OMD (24).

In the second study, 12 patients with idiopathic RLS (mean age 54.9 years, 7F, 5 M, mean duration of RLS symptoms 15.8 years) and 12 age- and sex-matched controls were given carbidopa/levodopa 50/200 at 11 p.m. and 11 a.m. with the administrations given at least a week apart (25). Blood was drawn before the administration of the drug (baseline) and every 15 min for up to 2 h after the administration of the drug. Hormonal levels were assayed for prolactin and growth hormone and cortisol. No differences were found between RLS patients and control at baseline in hormonal levels. As expected, L-dopa administration resulted in a suppression of prolactin levels in both groups. Within both groups, the decreases in plasma levels of prolactin were more pronounced at night than during the day (*p* < 0.05 at 90 min). At night, the decrease in plasma levels of prolactin after the L-dopa challenge was still more pronounced in RLS patients than controls with statistically significant differences 75 min after administration of the drug (*p* < 0.05). In the RLS group, the area under the curve of plasma prolactin levels at night showed a positive correlation to the PLM index as measured by polysomnography (*p* < 0.05). As also expected, L-dopa resulted in increases in growth hormone for both RLS patients and controls. Both groups had a greater increase at night than during the day. In addition, the increase in growth hormone was more pronounced after the night-time administration of the drug in the patients than in the controls (*p* < 0.05 at 45 min and *P* < 0.01 at 60, 75, and 90 min). No differences were found in any of the comparisons for serum cortisol with the L-dopa challenge. The authors postulate that their findings may reflect enhanced circadian variations in dopaminergic function in RLS and support a role for fluctuations in dopaminergic function as being pathogenetic to RLS with relatively asymptomatic daytimes and symptomatic night times (25).

In the third study, the authors looked at the circadian effects of longer-term dopaminergic treatment of RLS in eight previously untreated patients with idiopathic RLS (mean age 53 years, 3F, 5M, mean duration of RLS symptoms 26 years) (26). Patients underwent 3 weeks of treatment with carbidopa/levodopa 100/400. Dim light melatonin onset (DLMO), a marker of the circadian phase, was measured before and after treatment. When baseline was compared to treatment, an earlier DLMO was found after treatment (21:00 vs. 18:50, *p* < 0.05). The DLMO was even earlier in the RLS patients who had experienced augmentation vs. those who had not experienced augmentation (*p* < 0.05). Augmentation is a phenomenon that occurs when there is an initial improvement in RLS symptoms at night with dopaminergic treatment followed subsequently by the appearance of RLS symptoms at a slightly earlier time of day. With continued dopaminergic treatment, the symptoms may progressively go to yet even earlier times of the day. In this third study, a positive correlation was found between the change in DLMO and both sleep latency and the time of onset of RLS symptoms following L-Dopa treatment. The authors suggest that L-Dopa may exert chronobiotic effects in RLS (26). In other words, the timing of RLS symptoms could be under the control of the circadian pacemaker which in turn is under the control of dopamine. Dopamine tends to push RLS symptoms to an earlier circadian phase. Based upon extensive experience with the use of dopaminergic agents in the treatment of RLS, the higher the dopamine levels and the longer the dopamine exposure, the more the circadian pacemaker tends to phase advance RLS symptoms (26).

## Melatonin, bright light, and circadian rhythmicity of Restless Legs Syndrome/periodic limb movements in sleep

In the aforementioned study by Michaud et al., the authors noted that the acrophase of the profile of salivary melatonin occurred ~2 h before the acrophase of RLS leg discomfort and PLMS as opposed to the acrophase of body temperature and subjective vigilance that occurred after the acrophase of leg discomfort and PLMS (7). In addition, the authors point out that it is well-known that melatonin suppresses dopamine secretion in several areas of the mammalian central nervous system and that dopaminergic agents are well-established as one of the principal treatments of RLS. The authors reasoned, therefore, that in addition to its role as a circadian phase marker and its role in shifting the phase of circadian rhythms, that melatonin might play an additional role in the timing of the appearance of the leg discomfort of RLS and PLMS by suppressing dopamine (7). Other authors from the same group put this hypothesis to the test. In a study by Whittom et al., eight subjects with idiopathic RLS (mean age 53.3 years, range 38–63 years, 6F, 2M) were tested under three conditions: (a) at baseline; (b) after administration of melatonin 3 mg at 19:00 h; and (c) during bright light which, in addition to its role in shifting the timing of circadian rhythms, is a well-known suppressor of endogenous melatonin secretion. The bright light was administered at 3,000 lux from 19:00 until midnight (27). Procedures (b) and (c) were separated by 1 week and their order was reversed for half the subjects (27). A SIT test for each procedure was done two times, once in the early evening from 7:30 to 8:30 p.m. and before bedtime (from 11:00 p.m. to midnight). A visual analog scale was used to measure subjective leg discomfort and EMG was used to measure PLMW during the SIT. Results were partially supportive of the hypothesis of Michaud et al. (7, 27). There was a significant increase in PLMW with melatonin and a significant decrease in sensory leg discomfort with bright light. However, melatonin had no effect on leg discomfort and bright light had no effect on PLMW (27).

Moreover, in contradiction to some of these findings, a night-time polysomnographic study in nine patients with periodic limb movement disorder (PLMD) in the absence of RLS (mean age 57 years, range 40–71 years, 3F, 6M) showed that melatonin suppressed PLMS as measured by EMG (28). The overall motor activity also decreased at night as measured by actigraphy when the nights of the 14 days before and the 14 days during melatonin administration were compared (28).

Another study showed no difference in the 24-h urinary profile of 6 hydroxy-melatonin sulfate excretion between 15 patients with idiopathic RLS (mean age 52.1 years, 11F, 4M, mean duration of RLS symptoms 10.8 years) and 11 age- and sex-matched controls (29).

## Conclusion

Circadian studies indicate that RLS patients are worse later in the day or at night not just because they are sitting or lying down and RLS patients are worse sitting or lying down not just because it is later in the day or at night. Rather, the worsening later in the day or at night and the worsening on lying or sitting in RLS are related but separate phenomena (2–9). Circadian genes may play a role in the appearance and timing of RLS symptoms (10–12). The circadian rhythmicity of the hyperexcitability of the central (14–18) and peripheral (19, 20) nervous systems in RLS has been investigated as well as the circadian rhythmicity of cognition (21–23). The roles of dopamine (24–26), melatonin (26–29), and bright light (27) have been investigated in RLS in a circadian context.

## Future directions

Several issues remain as delineated in the following paragraphs:

All of the studies reported in this review are composed of RLS patients who have an average age of close to 40 years up to between 60 and 70 years. Thus, younger patients are largely under-represented in the studies and, as a result, several age-related questions remain. As an example, it has been noted that with time, RLS symptoms begin to appear earlier in the day in many patients. Some of this is thought to be caused by dopaminergic therapy as discussed earlier in the section on augmentation (26). However, there may be a significant number of patients who are not on dopaminergic therapy for which this is also the case. The pathophysiological changes that contribute to this and whether it represents a circadian advance in RLS symptoms with age need to be explored. Studying the circadian rhythmicity of RLS in younger adults and making the comparison to older adults or even in the same individuals over time may be helpful in this regard. Another age-related issue is that in children, circadian variability in RLS may not be apparent till later on. This is common enough for the International Classification of Sleep Disorders (ICSD-3) to state “Perhaps as a result of prolonged periods of sitting in class, two-thirds of children and adolescents with RLS report daytime leg sensations. Because of this, for the diagnostic criterion of worsening in the evening/night, it is important to compare the equal duration of sitting or lying down in the day to sitting or lying down in the evening/night. However, even with such comparisons, a significant subset of children does not report worsening at evening/night, yet meet all other diagnostic criteria and have supportive features for RLS, including positive family history.” (30). The reason why the circadian rhythmicity of RLS does not appear till later on needs to be further explored. Disease duration may also conceivably have an impact on the circadian rhythmicity of RLS but this has not been studied. The mean duration of RLS symptoms data was available for nine studies in this review and the RLS subjects had a disease duration of between 10.1 and 26 years.

All of the circadian studies in this review were conducted on patients with idiopathic RLS. Thus, studies on the circadian rhythmicity of RLS associated with other co-morbidities such as peripheral neuropathy are lacking. Moreover, although it has never been formally studied, all RLS whether it is idiopathic, familial, or associated with peripheral neuropathy, seems to follow the same circadian pattern based on clinical observation alone. The reasons for this circadian identity despite presumably different pathophysiology need to be explored as well. Certainly, some RLS that is diagnosed as idiopathic is instead comorbid with peripheral neuropathy since symptomatic small fiber sensory neuropathy may not be detected by neurological examination or routine EMG and nerve conductions studies but only by specialized and, not always readily available testing, such as nerve biopsy. However, not all patients with idiopathic RLS have subclinical peripheral neuropathy since many patients with idiopathic RLS have normal nerve biopsies as well. For example, in a study by Polydefkis et al., 22 consecutive RLS patients were studied with EMG and nerve conduction studies as well as peripheral nerve biopsy (31). In total, three patients had pure large fiber neuropathy, two had mixed large fiber neuropathy and small sensory fiber neuropathy, and three had isolated small sensory fiber neuropathy. In the other 14, the diagnostic studies were normal (31). More work needs to be done in this area.

In Whittom et al.'s study, bright light was not designed to look at whether the bright light could shift the circadian rhythmicity of the timing of appearance of leg sensory discomfort and PLMW in RLS, but rather to see whether bright light could affect leg discomfort and PLMW over a brief period in time (27). To further explore the circadian rhythmicity of RLS, SIT data should be collected over the entire 24-h circadian day. For the classic SIT, where patients are asked to lie perfectly still during the procedure, these data would include PLMW as measured by EMG or by specially programmed actigraphs with window settings for PLMs. For the modified SIT, where patients are allowed to move but only in response to sensory leg discomfort, the data would include leg movement as measured by routine actigraphy (6). For the classic or modified SIT, sensory discomfort would be measured by visual analog scales. These procedures should be performed before and after bright light exposure and the circadian phase should be determined in each instance by DLMO, continuous core body temperature monitoring, or both. If bright light shifts the signs and symptoms of RLS to a different circadian time in concert with circadian markers, this would more fully prove the circadian rhythmicity of RLS. Such studies need to be and should be conducted. Travel studies over several time zones could also be done where subjects with RLS are tested before, immediately after arrival, and several days after arrival. Subjects would need to be medication free to eliminate the confounding effects on symptoms.

Finally, in order to better define the role of circadian regulation, it is important to assess biomarkers of the internal timing system. Although endogenous melatonin has been considered the gold standard, it has practical challenges in the clinical setting, including dim light conditions and multiple sampling across 24 h. Recent developments of omics-based approaches and biomathematical predictive algorithms and modeling indicate that single- or two time points determination of circadian phase and alignment with sleep/wake timing can be accomplished in a laboratory and limited clinical settings. Thus, these advances in circadian biomarkers development could be applied to future RLS research (32–35).

## Author contributions

AW did the original draft of the manuscript. PZ reviewed and edited the manuscript. Both authors contributed to the article and approved the submitted version.
